# Transcriptome-wide association study of multiple myeloma identifies candidate susceptibility genes

**DOI:** 10.1186/s40246-019-0231-5

**Published:** 2019-08-20

**Authors:** Molly Went, Ben Kinnersley, Amit Sud, David C. Johnson, Niels Weinhold, Asta Försti, Mark van Duin, Giulia Orlando, Jonathan S. Mitchell, Rowan Kuiper, Brian A. Walker, Walter M. Gregory, Per Hoffmann, Graham H. Jackson, Markus M. Nöthen, Miguel Inacio da Silva Filho, Hauke Thomsen, Annemiek Broyl, Faith E. Davies, Unnur Thorsteinsdottir, Markus Hansson, Martin Kaiser, Pieter Sonneveld, Hartmut Goldschmidt, Kari Stefansson, Kari Hemminki, Björn Nilsson, Gareth J. Morgan, Richard S. Houlston

**Affiliations:** 1 Division of Genetics and Epidemiology, The Institute of Cancer Research, 15 Cotswold Road, Sutton, Surrey, SM2 5NG UK; 2 Division of Molecular Pathology, The Institute of Cancer Research, 15 Cotswold Road, Sutton, Surrey, SM2 5NG UK; 3 Department of Internal Medicine V, University of Heidelberg, 69117 Heidelberg, Germany; 4 German Cancer Research Center, 69120 Heidelberg, Germany; 5 Department of Hematology, Erasmus MC Cancer Institute, 3075 EA Rotterdam, The Netherlands; 6 Myeloma Institute for Research and Therapy, University of Arkansas for Medical Sciences, Little Rock, AR 72205 USA; 7 Clinical Trials Research Unit, University of Leeds, Leeds, LS2 9PH UK; 8 Institute of Human Genetics, University of Bonn, D-53127 Bonn, Germany; 9 Division of Medical Genetics, Department of Biomedicine, University of Basel, 4003 Basel, Switzerland; 10 Royal Victoria Infirmary, Newcastle upon Tyne, NE1 4LP UK; 11 Department of Genomics, Life & Brain Center, University of Bonn, D-53127 Bonn, Germany; 12 deCODE Genetics, Sturlugata 8, IS-101, Reykjavik, Iceland; 13 Hematology Clinic, Skåne University Hospital, SE-221 85 Lund, Sweden; 14 Hematology and Transfusion Medicine, Department of Laboratory Medicine, BMC B13, SE-221 84 Lund, Sweden; 15 Broad Institute, 7 Cambridge Center, Cambridge, MA 02142 USA

**Keywords:** Genome-wide association study, Gene expression, Multiple myeloma, Transcriptome-wide association study

## Abstract

**Background:**

While genome-wide association studies (GWAS) of multiple myeloma (MM) have identified variants at 23 regions influencing risk, the genes underlying these associations are largely unknown. To identify candidate causal genes at these regions and search for novel risk regions, we performed a multi-tissue transcriptome-wide association study (TWAS).

**Results:**

GWAS data on 7319 MM cases and 234,385 controls was integrated with Genotype-Tissue Expression Project (GTEx) data assayed in 48 tissues (sample sizes, *N* = 80–491), including lymphocyte cell lines and whole blood, to predict gene expression. We identified 108 genes at 13 independent regions associated with MM risk, all of which were in 1 Mb of known MM GWAS risk variants. Of these, 94 genes, located in eight regions, had not previously been considered as a candidate gene for that locus.

**Conclusions:**

Our findings highlight the value of leveraging expression data from multiple tissues to identify candidate genes responsible for GWAS associations which provide insight into MM tumorigenesis. Among the genes identified, a number have plausible roles in MM biology, notably *APOBEC3C*, *APOBEC3H*, *APOBEC3D*, *APOBEC3F*, *APOBEC3G*, or have been previously implicated in other malignancies. The genes identified in this TWAS can be explored for follow-up and validation to further understand their role in MM biology.

**Electronic supplementary material:**

The online version of this article (10.1186/s40246-019-0231-5) contains supplementary material, which is available to authorized users.

## Background

Multiple myeloma (MM) is the second most common hematologic malignancy in economically developed countries, and despite improvements in therapy, the disease essentially remains incurable. The aetiology of MM is poorly understood; however, the two- to four-fold increased risk of MM in relatives of patients has provided evidence for an inherited basis [1]. Direct evidence for inherited genetic susceptibility is provided by genome-wide association studies (GWAS), which have so far discovered 23 genomic regions harbouring risk variants for MM [2].

Consistent with findings from many different cancer GWAS, bar a few notable exceptions, the functional variants and target susceptibility genes at the MM risk regions are yet to be identified. Knowledge of the causal genes responsible for defining disease predisposition is important in furthering our understanding of MM tumorigenesis and has the potential to inform the development of novel therapeutic strategies [3]. While most GWAS risk variants map to non-coding regions of the genome, they are enriched for variants correlated with gene expression levels [4, 5]. Exploiting this characteristic, the integration of GWAS signals with expression quantitative trait loci (eQTLs) has implicated *ELL2* and *CDCA7L* as the risk genes likely to be responsible for the 5q15 and 7p15.3 MM associations, respectively [6–9]. The high frequency of eQTLs coupled with linkage disequilibrium (LD) across regions can, however, make disentangling the risk genes from spurious co-localization at the same region problematic.

Transcriptome-wide association studies (TWAS) have been proposed as a strategy to identify risk genes underlying complex traits [10]. This approach imputes genetic data from GWAS using reference sets of weights generated from eQTL data, before correlating this genetic component of gene expression with the phenotype of interest. Since TWAS aggregates the effects of multiple variants into a single testing unit, and facilitates prioritisation of genes at known risk regions for functional validation, it potentially also affords increased study power to identify new risk regions.

While MM is caused by the clonal expansion of malignant plasma cells, if a TWAS is to be based on expression data from a single cell deciding on the most appropriate source is inherently problematic [11]. Utilising eQTL data from tumours is complicated by copy number alterations and essentially represents terminal stage in disease progression. Moreover, the effect of any risk allele may be acting at the level of the tumour micro-environment [12]. Studies have shown that eQTLs strongly enriched in GWAS signals are not necessarily specific to the eQTL discovery tissue [5]. Taking advantage of this principle allows a multi-tissue TWAS to be conducted integrating expression across multiple tissues, thereby leveraging information on shared eQTLs for candidate gene discovery [13].

Herein, we report a multi-tissue TWAS to prioritise candidate causal genes at known risk regions for MM and search for new risk regions. Specifically, we have analysed gene expression data from 48 tissue panels measured in 8756 individuals in conjunction with summary association statistics on 7319 MM cases and 234,385 controls of European descent. We identify 108 genes at 13 loci associated with MM risk and provide additional evidence of a potential role for a number of genes dysregulated in MM tumorigenesis.

## Results

**Fig. 1:**
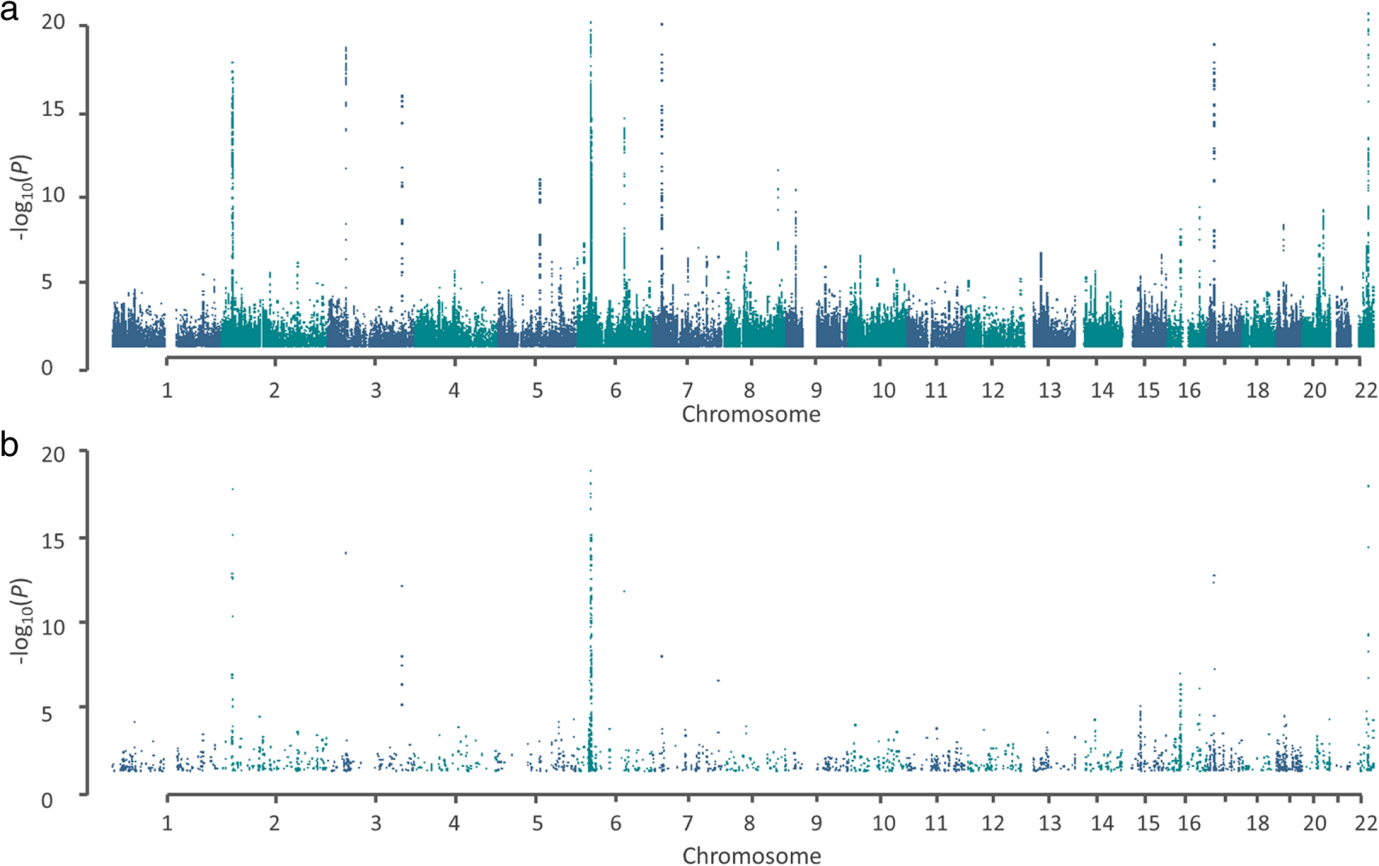
Manhattan plots of gene genomic co-ordinates against –log_10_(*P* value) of GWAS and TWAS association statistics. **a** GWAS association statistics. **b** TWAS association statistics

We evaluated the association between predicted gene expression levels and MM risk using MetaXcan with summary statistics for GWAS SNPs in 7319 MM cases and 234,385 controls. In total, the expression levels of 25,520 genes across 48 tissues were tested for an association with MM risk. Quantile-quantile plots of TWAS association statistics did not show evidence of systematic inflation (Additional file 1: Figure S1). Figure 1 shows Manhattan plots for respective GWAS and TWAS associations.

Applying a Bonferroni threshold, we identified 108 genes at 13 independent regions associated with MM (Table 1, Additional file 1: Table S1). All identified genes except those localising to the HLA region on chromosome 6p21 were within 1 Mb of previously reported MM risk SNPs. For all loci, except those in the HLA region, association signals were abrogated after adjusting for the top risk SNP, consistent with variation in expression of the identified gene being functionally related to the MM risk association. The complex LD patterns within the HLA region make deconvolution of significant results within the region difficult [14, 15]; therefore, our principal focus was confined to 31 genes at 12 loci outside 6p21.

**Table 1:** Genes significantly associated with risk of multiple myeloma

Locus	Gene	*P* value	*N*/*N*_indep_	*Z*-score min	*Z*-score max	*Z*-score mean	*Z*-score s.d.	SNP adjusting for	*P* value after SNP adjustment
16p11.2	*QPRT*	1.01 × 10^−7^	17/8	− 2.73	3.04	− 0.59	1.63	rs13338946	0.15
16p11.2	*RNF40*	4.02 × 10^−7^	24/3	0.05	5.68	4.67	1.48	rs13338946	0.89
16p11.2	*PRR14*	4.28 × 10^−7^	2/2	− 5.38	− 0.20	− 2.79	3.66	rs13338946	0.34
16p11.2	*C16orf93*	8.07 × 10^−7^	13/5	− 5.74	− 0.34	− 4.59	1.73	rs13338946	0.24
16p11.2	*RP11-2C24.5*	1.54 × 10^−6^	5/5	− 5.64	4.43	− 0.58	3.80	rs13338946	0.73
16p11.2	*PRSS53*	1.71 × 10^−6^	16/8	− 5.19	3.68	− 1.04	2.71	rs13338946	0.79
16q23.1	*RFWD3*	7.71 × 10^−7^	34/7	− 3.41	6.35	2.51	3.26	rs7193541	0.47
17p11.2	*TBC1D27*	1.95 × 10^−13^	6/6	− 1.91	4.19	0.51	2.16	rs34562254	0.89
17p11.2	*USP32P1*	4.88 × 10^− 13^	3/3	− 7.29	2.80	−1.36	5.27	rs34562254	0.01
17p11.2	*PEMT*	5.65 × 10^−8^	14/7	− 1.74	5.43	1.36	1.93	rs34562254	0.01
22q13.1	*APOBEC3C*	1.10 × 10^−18^	21/8	− 8.93	0.24	− 4.09	2.21	rs139402	0.13
22q13.1	*APOBEC3H*	4.28 × 10^−15^	7/5	− 5.45	7.92	− 0.95	4.38	rs139402	0.76
22q13.1	*FAM83F*	4.65 × 10^−10^	11/8	− 4.25	2.56	− 0.48	2.01	rs139402	1.1 × 10^−4^
22q13.1	*APOBEC3D*	6.2 × 10^−10^	29/7	− 8.38	− 0.85	− 4.15	1.56	rs139402	0.04
22q13.1	*APOBEC3F*	5.15 × 10^−9^	5/4	− 6.34	6.15	1.09	5.07	rs139402	0.13
22q13.1	*APOBEC3G*	1.81 × 10^−7^	43/2	0.36	6.57	4.94	1.17	rs139402	0.17
2p23.3	*KIF3C*	1.65 × 10^−18^	6/6	− 9.40	4.35	− 1.19	4.50	rs7577599	1.4 × 10^−9^
2p23.3	*EPT1*	8.37 × 10^−16^	9/9	− 1.76	6.00	1.30	2.72	rs7577599	2.1 × 10^−5^
2p23.3	*CENPO*	1.48 × 10^−13^	12/8	− 6.60	2.22	− 0.05	2.57	rs7577599	6.1 × 10^−8^
2p23.3	*DNMT3A*	2.44 × 10^−13^	8/8	− 2.89	7.96	1.94	3.07	rs7577599	0.01
2p23.3	*AC010150.1*	2.90 × 10^−13^	4/4	− 0.88	7.89	1.61	4.20	rs7577599	8.9 × 10^−10^
2p23.3	*PTGES3P2*	4.46 × 10^−11^	7/5	− 4.23	2.03	− 2.46	2.08	rs7577599	1.1 × 10^−4^
2p23.3	*DTNB*	1.16 × 10^−7^	11/10	− 3.88	5.78	0.36	2.38	rs7577599	3.1 × 10^−3^
2p23.3	*DNAJC27*	1.74 × 10^−7^	8/8	− 0.74	4.52	1.95	1.58	rs7577599	0.11
3p22.1	*ULK4*	9.01 × 10^−15^	43/6	0.90	8.89	6.60	2.24	rs6599192	0.85
3q26.2	*MYNN*	7.84 × 10^−13^	6/6	− 7.91	1.58	− 1.66	3.32	rs10936600	0.17
3q26.2	*LRRIQ4*	9.63 × 10^−9^	3/2	− 5.94	− 0.88	− 4.25	2.92	rs10936600	0.03
3q26.2	*LRRC34*	3.35 × 10^−8^	21/2	3.97	6.47	5.12	0.66	rs10936600	0.82
3q26.2	*ACTRT3*	4.28 × 10^−7^	4/4	− 0.94	5.80	1.56	2.94	rs10936600	0.48
6q21	*ATG5*	1.55 × 10^−12^	4/4	0.93	5.89	3.72	2.41	rs9372120	0.07
7p15.3	*CDCA7L*	9.61 × 10^−9^	8/8	− 3.11	4.61	1.12	2.42	rs75341503	0.23
7q36.1	*CHPF2*	2.53 × 10^−7^	6/6	− 2.01	2.13	0.40	1.49	rs7781265	0.06

Excludes associations found in the HLA region. s.d., standard deviation. Detailed are the S-MultiXcan *P* values for association between gene expression MM, and the corresponding *Z*-scores quantifying this relationship (e.g. a positive score indicates increased gene expression increases risk). *N* and *N*_indep_ indicate the total number of single-tissue results used for S-MultiXcan analysis and the number of independent components after singular value decomposition, respectively

For many loci, our TWAS findings support the involvement of a number of genes that have previously been implicated in defining MM [2, 16–19]. Specifically, single-gene associations were identified at 3p22.1 (*ULK4*), 6q21 (*ATG5*), 7p15.3 (*CDCA7L*), 7q36.1 (*CHPF2*) and 16q23.1 (*RFWD3*). However, at a number of regions, our analysis identified multiple significant genes, notably, 2p23.3 (*KIF3C*, *EPT1*, *CENPO*, *DTNB*, *DNM3TA*, *PTGES3P2*, *DNAJC27*), 3q26.2 (*MYNN*, *LRRC34*, *LRRIQ4*, *ACTRT3*), 16p11.2 (*QPRT*, *RNF40*, *PRR14*, *C16orf93*, *RP11-2C24.5*, *PRSS53*) and 17p11.2 (*TBC1D27*, *USP32P1*, *PEMT*). A complete list of novel genes identified at known GWAS risk loci is provided in Additional file 1: Table S2.

**Fig. 2:**
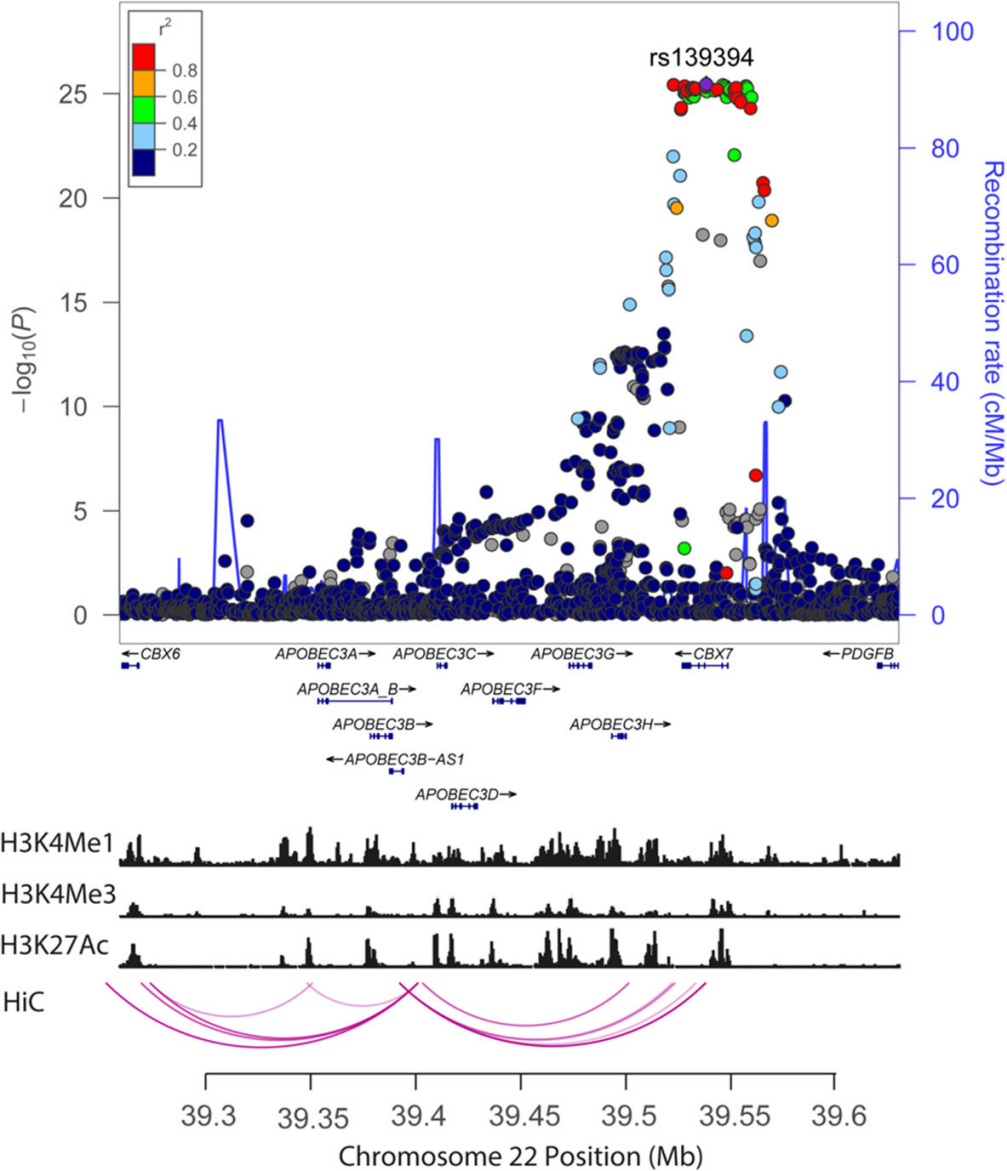
Regional plot of association results at 22q13 in MM alongside recombination rates and histone marks in GM12878. Plot shows discovery association results of both genotyped and imputed SNPs in the GWAS samples and recombination rates. −log10 *P* values (*y* axes) of the SNPs are shown according to their chromosomal positions (*x* axes). The colour of each symbol reflects the extent of LD with the top genotyped SNP. Genetic recombination rates, estimated using HapMap samples from Utah residents of western and northern European ancestry (CEU), are shown with a blue line. Physical positions are based on NCBI build 37 of the human genome. Also shown are the relative positions of GENCODE v19 genes mapping to the region of association. Below the association plot are the relative positions of GENCODE v19 genes mapping to the region of association and the histone marks and chromatin loops for lymphoblastoid cell line, GM12878

Interestingly, several of the *APOBEC* genes were identified at 22q13.1. These genes localise within a distinct LD block adjacent to the one to which the sentinel GWAS risk SNPs maps (Fig. 2). We sought to gain insight into the potential for genome-wide significant SNPs in 22q13.1 in to influence regulation via a *cis*-regulatory enhancer, by mapping looping interaction and histone modifications in the lymphoblastoid cell line GM12878, which was chosen as a model for early B cell differentiation, with negligible genetic and phenotypic abnormalities [20]. We found evidence of enhancer marks and looping interactions from SNPs in 22q13.1 to *APOBEC* genes (Fig. 2), highlighting active chromatin and spatial proximity present in this region, necessary to mediate gene expression [21]. No significant genes were identified at 12 reported MM risk regions (2q31.1, 5q15, 5q23.2, 6p22.3, 7q22.3, 7q31.33, 8q24.21, 9p21.3, 10p12.1, 17p11.2, 19p13.1, 20q13.1).

## Discussion

In this large TWAS involving 7319 MM cases of European ancestry, we identified genetically predicted expression levels in 108 genes associated with MM risk. Of these, there were 94 genes located in eight regions that, although mapping within 1 Mb of a MM risk locus had not previously been considered as a candidate gene for that locus.

Our findings provide further support for a number of the genes previously implicated by GWAS whose expression influences the risk of developing MM, including *CDCA7L* at 7p15.3, which has been functionally validated. At 7p15.3, rs4487645 resides in an enhancer of c-Myc-interacting CDCA7L and increases IRF4 binding, affecting MM proliferation [7]. Furthermore, *ULK4* at 3p22.1, *ATG5* at 6q21 and *RFWD3* at 16q23 have been identified here and implicated previously. Additionally, our TWAS implicates new genes at known risk regions, notably *APOBEC3C, APOBEC3D, APOBEC3F, APOBEC3G and APOBEC3H* at 22q13.1 as playing a role in defining MM predisposition. Aberrant APOBEC cytidine deaminase activity has been shown to correlate with an increased mutational burden and is a recognised feature of MM, caused by triggering DNA mutation through dC deamination [22–24]. Furthermore, *KIF3C,* identified at 2p23.3, is a gene which regulates microtubule dynamics and has been previously implicated in breast cancer [25, 26]. Also at 2p23.3, this analysis identified *CENPO*, a gene involved in cell cycle progression via regulation of kinetochore assembly [27]. At 16p11.2, *RNF40* is a promising candidate for MM susceptibility due to its role in double-strand break repair during homologous recombination (HR) and class switch recombination [28, 29]. This gene has also been implicated in colorectal cancer [30]. A further candidate at this locus, *QPRT* has been demonstrated to confer resistance to chemotherapy and radiotherapy when studied in glioma and leukaemia [31, 32]. As such, genes identified within this TWAS build upon previously suggested candidate disease mechanisms which may confer MM predisposition [2], including anti-apoptotic effects, roles in DNA double-strand break repair and cell cycle regulation. Furthermore, many of the genes identified have been previously investigated in vitro for their roles in cancer and this adds further support as plausible candidate genes for MM predisposition [24, 26, 30–32].

6p21.33, which encodes much of the major histocompatibility complex, is an especially gene rich region. As well as the class I HLA-A and class II genes HLA-DQA1 and HLA-DRB1/5, multiple genes localise to the region including *TCF19* which encodes the cell cycle progression and proliferation transcription factor 19 [33, 34]. Complex LD patterns within this region make deconvolution of significant results within the region inherently problematic [14]. Additional work is required to reveal the contribution of genes in this region to MM development.

A number of previously reported MM risk regions were not implicated in our TWAS. At some regions such as 5q15, the high tissue specificity associated with the causal gene *ELL2* [6] may not be best modelled herein. At other loci, it is less obvious why an association was not detected. Speculatively, models at earlier developmental stages may yield greater insights at these loci, especially if they are influencing differentiation along B cell lineages. Additionally, other mechanistic effects may explain the functional basis of such loci, including methylation and splicing.

The increasing appreciation that regulation of gene expression forms the mechanistic basis of many GWAS risk regions makes the TWAS an attractive approach to identify causal genes. Traditionally, studies have only tended to consider an eQTL and risk SNP to overlap if they are in linkage at a specified threshold. This is, however, conservative as multiple local SNPs may independently contribute to risk. Furthermore, stipulating genome-wide significance thresholds for the GWAS signal (i.e. *P* < 5 × 10^−8^) and linkage strength (i.e. LD > 0.5) between pairs of SNPs for evidence of expression influencing risk, constrains study power. The TWAS approach is essentially agnostic as it jointly considers all SNPs in the region, regardless of reported GWAS association strength. There are, however, limitations to TWAS. Firstly, TWAS is based on fitting predictive linear models of gene expression based on local genotype data, followed by prediction into large cohorts and subsequent association testing; therefore, it does not capture total expression which includes environmental and technical components [35]. Secondly, TWAS will also lose power if gene expression is a nonlinear function of local SNPs, or when trans (or distal) regulation is a major determinant of expression levels.

All conclusions from our TWAS come with several caveats. While TWAS associations are consistent with models of gene expression level influencing MM risk, we acknowledge the possibility of confounding. Imputed gene expression levels are generated from weighted linear combinations of SNPs, and many of which may tag non-regulatory mechanisms driving risk and result in inflated association statistics. Inevitably, despite addressing LD, since genes with eQTLs are common, associations may be the result of chance co-localization between eQTLs and MM risk.

Our ability to identify gene expression significantly associated with MM risk in this TWAS may be affected by tissue specificity. On the basis of the power calculation, our TWAS analysis had only 80% power to detect an odds ratio of ~ 1.1 for MM risk per one standard deviation increase (or decrease) in the expression level of a gene whose *cis*-heritability is 60% respectively in EBV-transformed lymphocytes (Additional file 1: Figure S2), which we used as a proxy for plasma cells. In light of abundant shared *cis*-regulation of expression across tissues, by combining data, we would expect any model to yield greater power as the number of tissues increases in which a variant is functional. Hence, we aimed to robustly capture genetically regulated gene expression using a large sample size.

## Conclusions

Our findings highlight the value of integrating expression with GWAS to prioritise candidate causal genes. A number of identified genes have plausible roles in MM tumourigenesis (e.g. *APOBEC, RNF40*) or have been previously implicated in other malignancies (e.g. *QPRT*). The genes identified in this TWAS can be explored for follow-up and validation to further understand their role in MM biology.

## Methods

### GWAS data

MM genotyping data were derived from the most recent meta-analysis of 7 GWAS datasets totalling 7319 cases and 234,385 controls of European descent. After imputation, these related > 3.5 million genetic variants to MM. Comprehensive details of the genotyping and quality control of these GWAS have been previously reported [2, 16–19] and are summarised in Additional file 1: Tables S3 and S4.

### Association analysis of predicted gene expression with myeloma risk

Associations between predicted gene expression and MM risk were examined using MetaXcan [10], which combines GWAS and eQTL data, accounting for LD-confounded associations. Briefly, genes likely to be disease-causing were prioritised using S-PrediXcan [10] which uses GWAS summary statistics and pre-specified weights to predict gene expression, given co-variances of SNPs. SNP weights and their respective covariance in 48 tissues from 80 to 491 individuals were obtained from predict.db (http://predictdb.org/), which is based on GTEx version 7 eQTL data [36]. A full list of the sample count by tissue can be found at https://gtexportal.org/home/tissueSummaryPage. To combine S-PrediXcan data across the different tissues taking into account tissue-tissue correlations, we used S-MultiXcan [13].

To determine if associations between genetically predicted gene expression and MM risk were influenced by variants previously identified by GWAS, we performed conditional analyses adjusting for sentinel GWAS risk SNPs (Additional file 1: Table S5) using GCTA-COJO [37]. Adjusted output files were provided as the input GWAS summary statistics for S-PrediXcan analyses as above. To account for multiple comparisons, we considered a Bonferroni-corrected *p* value threshold of 1.96 × 10^−6^ (i.e. 0.05/25,520 genes) as being statistically significant.

### Regulatory annotation

To map risk SNPs to interactions involving promoter contacts and identify genes involved in MM susceptibility at the 22q13.1 locus, we analysed previously published promoter capture Hi-C data on the GM12878 downloaded from the ArrayExpress database, accession code E-MTAB-2323 cell line as a model B cell [38]. Reads from technical replicates were combined before processing and valid pairs were identified using HICUP [39]. Two biological replicates were analysed to assure reproducibility and significant interactions were determined using CHiCAGO [40]. ChIP-Seq on H3K4Me1, H3K4Me3, and H3K27Ac in GM12878 were from the ENCODE project (ENCODE Project Consortium, 2012).

### Statistical power for association tests

To estimate the power of our TWAS to identify associations, we performed a simulation analysis adopting a similar methodology to Wu et al. [41] We set the number of cases and controls as 7319 and 234,385, respectively. An estimate of the population prevalence of MM was obtained from Cancer Research UK (https://www.cancerresearchuk.org). We generated the gene expression levels from the empirical distribution of gene expression levels in GTEx normalised expression dataset for each tissue. We calculated statistical power at *P* < 1.96 × 10^−6^, corresponding to the TWAS genome-wide significance level, according to various *cis*-heritability (*h*^*2*^) thresholds that are assumed to be equivalent to gene expression prediction models (*R*^*2*^). The results are based on 1000 replicates.

## Additional file

Additional file 1: **Table S1.** Genes significantly associated with risk of multiple myeloma. **Table S2.** New and previously implicated^1-5^ genes at each genome wide significant multiple myeloma locus**. Table S3.** Quality control filters applied to samples from the seven published GWAS. **Table S4.** Quality control filters applied to SNPs from each GWAS. **Table S5.** MM GWAS risk SNPs. **Figure S1.** Quantile-Quantile Plots of –log10(*P*-value) associations. **Figure S2.** TWAS power plot in EBV-transformed lymphocytes. (DOCX 1515 kb)

## Data Availability

Details and availability of SNP genotyping data that support the findings of this study have been previously published [2, 16–19].
