# Supplementary material for: Transcriptome-wide association study of multiple myeloma identifies candidate susceptibility genes
Source: Hum Genomics. 2019 Aug 20;13:37. doi: 10.1186/s40246-019-0231-5 (PMC6700979; doi:10.1186/s40246-019-0231-5)
Supplement: Supplementary file 1 — Table S1. Genes significantly associated with risk of multiple myeloma. Table S2. New and previously implicated1-5 genes at each genome wide significant multiple myeloma locus. Table S3. Quality control filters applied to samples from the seven published GWAS. Table S4. Quality control filters applied to SNPs from each GWAS. Table S5. MM GWAS risk SNPs. Figure S1. Quantile-Quantile Plots of –log10(P-value) associations. Figure S2. TWAS power plot in EBV-transformed lymphocytes. (DOCX 1515 kb) [file 40246_2019_231_MOESM1_ESM.docx]

**SUPPLEMENTARY INFORMATION**

### Transcriptome-wide association study of multiple myeloma identifies candidate susceptibility genes

**Went *et al.***

**Supplementary Table 1: Genes significantly associated with risk of multiple myeloma.** Includes associations seen in the HLA region (6p21.32-33,6p22.1). s.d., standard deviation. Detailed are the S-MultiXcan *P*-value for association between gene expression and the corresponding Z-scores quantifying this relationship (*e.g.* a positive score indicates increased gene expression increases risk). N and N_indep_ indicate the total number of single-tissue results used for S-MultiXcan analysis and the number of independent components after singular value decomposition, respectively.

| **SNP** | **Locus** | **bp(b37)** | **Newly implicated genes** | **Previously identified genes** | **Study** |
| --- | --- | --- | --- | --- | --- |
| rs7577599 | 2p23.3 | 25613146 | KIF3C, EPT1, CENPO, DNMT3A, AC010150.1, PTGES3P2, DNAJC27 | *DTNB* | Broderick *et al* 2011 |
| rs4325816 | 2q31.1 | 174808899 |  | *SP3* | Went *et al* 2018 |
| rs6599192 | 3p22.1 | 41992408 |  | *ULK4* | Broderick *et al* 2011 |
| rs10936600 | 3q26.2 | 169514585 | LRRIQ4 | *TERC, ACTRT3, MYNN, LRRC34, GPR160, LRRC31, MYNN, PDCD10, SERPINI1, SEC62, SAMD7, SEC62-AS1, SKIL, PHC3, PDCD10* | Chubb *et al* 2013, Went *et al* 2018 |
| rs1423269 | 5q15 | 95255724 |  | *ELL2, VPS13C* | Swaminathan *et al*, Li *et al* 2017 |
| rs6595443 | 5q23.2 | 122743325 |  | *CEP120, SNX2, SNX24* | Went *et al* 2018 |
| rs34229995 | 6p22.3 | 15244018 |  | *JARID2* | Mitchell *et al* 2016 |
| rs3132535 | 6p21.3 | 31116526 |  | *PSORS1C1, CCHCR1, CDSN, TCF19, POU5F1* | Chubb *et al* 2013, Went *et al* 2018 |
| rs9372120 | 6q21 | 106667535 |  | *ATG5, PREP, PRDM1* | Micthell *et al* 2016, Went *et al* 2018 |
| rs4487645 | 7p15.3 | 21938240 |  | *DNAH11 CDCA7L* | Broderick *et al* 2011, Li *et al* 2016 |
| rs17507636 | 7q22.3 | 106291118 |  | *CCDC71L* | Went *et al* 2018 |
| rs58618031 | 7q31.33 | 124583896 |  | *POT1, ASB15, IQUB, WASL* | Went *et al* 2018 |
| rs7781265 | 7q36.1 | 150950940 |  | *ABCF2 CHPF2 SMARCD3 ASIC3 ATG98* | Mitchell *et al* 2016 |
| rs1948915 | 8q24.21 | 128222421 |  | *CASC11, MYC* | Mitchell *et al* 2016, Went *et al* 2018 |
| rs2811710 | 9p21.3 | 21991923 |  | *CDKN2A, MTAP, CDKN2B-AS1* | Mitchell *et al* 2016 |
| rs2790457 | 10p12.1 | 28856819 |  | *WAC, LYZL1, MASTL, YME1L1* | Mitchell *et al* 2016, Went *et al* 2018 |
| rs13338946 | 16p11.2 | 30700858 | QPRT, RNF40, RP11-2C24.5, C16orf93 | *PRR14, FBRS, SRCAP, DCTPP1, DOC2A, FBXL19, GDPD3, ITGAL, MYLPF, PPP4C, SEPHS2, SEPT1, TBC1D10B, ZNF48, ZNF771* | Went *et al* 2018 |
| rs7193541 | 16q23.1 | 74664743 |  | *RFWD3, GLG1, HSPE1P, CFDP1, PSMD7, GABARAPL2, NPIPL2* | Mitchell *et al* 2016 |
| rs34562254 | 17p11.2 | 16842991 | PEMT, USP32P1, TBC1D27 | *TNFRSF13B* | Chubb *et al* 2013 |
| rs11086029 | 19p13.11 | 16438661 | N/A | *KLF2* | Went *et al* 2018 |
| rs6066835 | 20q13.13 | 47355009 | N/A | *PREX1, ARFGEF2* | Mitchell *et al* 2016, Went *et al* 2018 |
| rs138747 | 22q13.1 | 35700488 | N/A | *CRYBB1, HMOX1, APOL3, TOM1, LARGE, FBXO7, HMGXB4, RASD2, MB* | Swaminathan *et al* 2015, Went *et al* 2018 |
| rs139402 | 22q13.1 | 39546145 | APOBEC3C, APOBEC3D, APOBEC3F, APOBEC3G, APOBEC3H, FAM83F | *CBX7, APOBEC3B-AS1, RPL3* | Chubb *et al* 2013, Went *et al* 2018 |

**Supplementary Table 2:** New and previously implicated^1-5^ genes at each genome wide significant multiple myeloma locus.

|  |  |  |  |  |  |  |  |  |  |  |  |  |  |  | |
| --- | --- | --- | --- | --- | --- | --- | --- | --- | --- | --- | --- | --- | --- | --- | --- |
|  | **UK** | | **Sweden/Norway** | | **Germany** | | **Netherlands** | | **USA** | | **Iceland** | | **OncoArray** | | |
|  | **Cases** | **Controls** | **Cases** | **Controls** | **Cases** | **Controls** | **Cases** | **Controls** | **Cases** | **Controls** | **Cases** | **Controls** | **Cases** | **Controls** | |
| Pre-QC | 2,329 | 5,199 |  |  | 1,512 | 2,107 | 608 | 2,669 | 1,076 | 2,234 |  |  | 931 | 7,519 |  |
|  |  |  |  |  |  |  |  |  |  |  |  |  |  |  | |
| Sex discrepancy | 10 | 0 |  |  | 1 | 0 | 0 | 0 | 0 | 0 |  |  | 6 | 8 | |
| Call rate fail | 1 | 0 |  |  | 0 | 0 | 2 | 0 | 0 | 4 |  |  | 1 | 1 | |
| Heterozygosity rate | NA | NA |  |  | NA | NA | 7 | 0 | 9 | 2 |  |  | 5 | 7 | |
| Related Individuals | 2 | 2 |  |  | 0 | 0 | 0 | 0 | 1 | 0 |  |  | 3 | 68 | |
| Non-European Ancestry | 34 | 0 |  |  | 3 | 0 | 44 | 0 | 286 | 369 |  |  | 44 | 364 | |
|  |  |  |  |  |  |  |  |  |  |  |  |  |  |  | |
| Post-QC | 2,282 | 5,197 | 1,714 | 10,391 | 1,508 | 2,107 | 555 | 2,669 | 780 | 1,857 | 480 | 212,164 | 878 | 7,083 | |

**Supplementary Table 3: Quality control filters applied to samples from the seven published GWAS.** Samples were excluded due to call rate (<95% or failed genotyping), ancestry (principle components analysis or other samples reported to be not of white, European descent), relatedness (any individuals found to be duplicated or related within or between data sets through IBS) or sex discrepancy. All studies have been previously reported in their entirety with comprehensive details on QC^1^.

|  | **UK** | **Sweden/Norway** | **Germany** | **Netherlands** | **USA** | **Iceland** | **OncoArray** |
| --- | --- | --- | --- | --- | --- | --- | --- |
|  |  |  |  |  |  |  |  |
| Pre-QC | 409,429 |  | 401,405 | 646,124 | 296,998 |  | 459,068 |
| Call rate fail | 997 |  | 113 | 6,523 | 4 |  | 6,851 |
| HWE fail | 7 |  | 0 | 18,104 | 171 |  | 12 |
| MAF < 0.01 | 3 |  | 1 | 0 | 9,151 |  | 73,239 |
| Post-QC | 408,422 |  | 401,291 | 621,497 | 287,672 |  | 378,966 |
| Imputed (filtered) | 8,517,071 | 7,182,761 | 8,282,831 | 8,628,799 | 8,085,846 | 10,291,845 | 8,309,872 |

**Supplementary Table 4: Quality control filters applied to SNPs from each GWAS.** Studies have been previously reported in their entirety with comprehensive details on QC^1^.

| **SNP** | **Locus** | **Chr.** | **Pos. (b37)** | **Risk Allele** | **RAF** | **OR** | ***P*_meta_** | ***I*^2^** |
| --- | --- | --- | --- | --- | --- | --- | --- | --- |
| rs7577599 | 2p23.3 | 2 | 25613146 | T | 0.81 | 1.23 | 1.29×10^-18^ | 0 |
| rs4325816 | 2q31.1 | 2 | 174808899 | T | 0.77 | 1.12 | 7.37×10^-9^ | 9 |
| rs6599192 | 3p22.1 | 3 | 41992408 | G | 0.16 | 1.26 | 4.96×10^-20^ | 0 |
| rs10936600 | 3q26.2 | 3 | 169514585 | A | 0.75 | 1.20 | 1.20×10^-16^ | 0 |
| rs1423269 | 5q15 | 5 | 95255724 | A | 0.75 | 1.16 | 8.30×10^-12^ | 23 |
| rs6595443 | 5q23.2 | 5 | 122743325 | T | 0.43 | 1.11 | 1.20×10^-8^ | 0 |
| rs34229995 | 6p22.3 | 6 | 15244018 | G | 0.02 | 1.36 | 5.60×10^-8^ | 0 |
| rs3132535 | 6p21.3 | 6 | 31116526 | A | 0.29 | 1.21 | 6.00×10^-21^ | 0 |
| rs9372120 | 6q21 | 6 | 106667535 | G | 0.21 | 1.19 | 2.40×10^-15^ | 0 |
| rs4487645 | 7p15.3 | 7 | 21938240 | C | 0.65 | 1.24 | 2.80×10^-28^ | 0 |
| rs17507636 | 7q22.3 | 7 | 106291118 | C | 0.74 | 1.12 | 9.20×10^-9^ | 50 |
| rs58618031 | 7q31.33 | 7 | 124583896 | T | 0.72 | 1.12 | 2.73×10^-8^ | 0 |
| rs7781265 | 7q36.1 | 7 | 150950940 | A | 0.12 | 1.22 | 4.82×10^-10^ | 49 |
| rs1948915 | 8q24.21 | 8 | 128222421 | C | 0.32 | 1.15 | 2.53×10^-12^ | 26 |
| rs2811710 | 9p21.3 | 9 | 21991923 | C | 0.63 | 1.14 | 3.64×10^-11^ | 0 |
| rs2790457 | 10p12.1 | 10 | 28856819 | G | 0.73 | 1.11 | 2.66×10^-6^ | 0 |
| rs13338946 | 16p11.2 | 16 | 30700858 | C | 0.26 | 1.15 | 1.02×10^-13^ | 26 |
| rs7193541 | 16q23.1 | 16 | 74664743 | T | 0.58 | 1.12 | 3.68×10^-10^ | 34 |
| rs34562254 | 17p11.2 | 17 | 16842991 | A | 0.10 | 1.30 | 1.18×10^-19^ | 29 |
| rs11086029 | 19p13.11 | 19 | 16438661 | T | 0.24 | 1.14 | 6.79×10^-11^ | 42 |
| rs6066835 | 20q13.13 | 20 | 47355009 | C | 0.08 | 1.23 | 6.58×10^-10^ | 38 |
| rs138747 | 22q13.1 | 22 | 35700488 | A | 0.66 | 1.21 | 2.58×10^-8^ | 0 |
| rs139402 | 22q13.1 | 22 | 39546145 | C | 0.44 | 1.22 | 3.84×10^-26^ | 56 |

**Supplementary Table 5:** MM GWAS risk SNPs. RAF, risk allele frequency; *P*_meta_, *P*-value for fixed effects meta-analysis; *I*^2^, heterogeneity index (0–100). RAF is based on the UK cohort control series, with the exception of rs138747, which is sourced from the 1000 Genomes Project^6^.

**Supplementary Table 3: Summary of genotyping results for all 23 MM risk SNPs.** RAF, risk allele frequency; *P*_trend_, *P*-value for trend, via logistic regression; *P*_meta_, *P*-value for fixed effects meta-analysis; *I*^2^, heterogeneity index (0–100). RAF are based on the UK cohort control series, with the exception of rs138747, which is sourced from the 1000 Genomes Project.


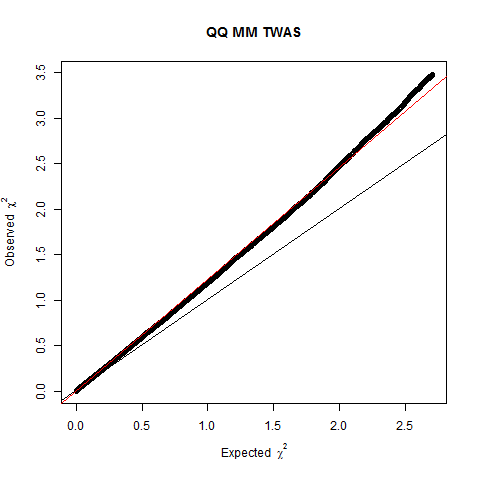

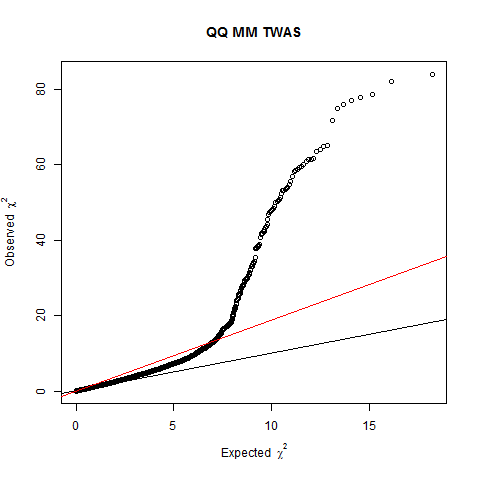


λ=1.22

λ=1.88

**Supplementary Figure 1: Quantile-Quantile Plots of –log10(*P*-value) associations.** (top) TWAS for MM; (bottom) TWAS for MM (lower 90% of associations).

**
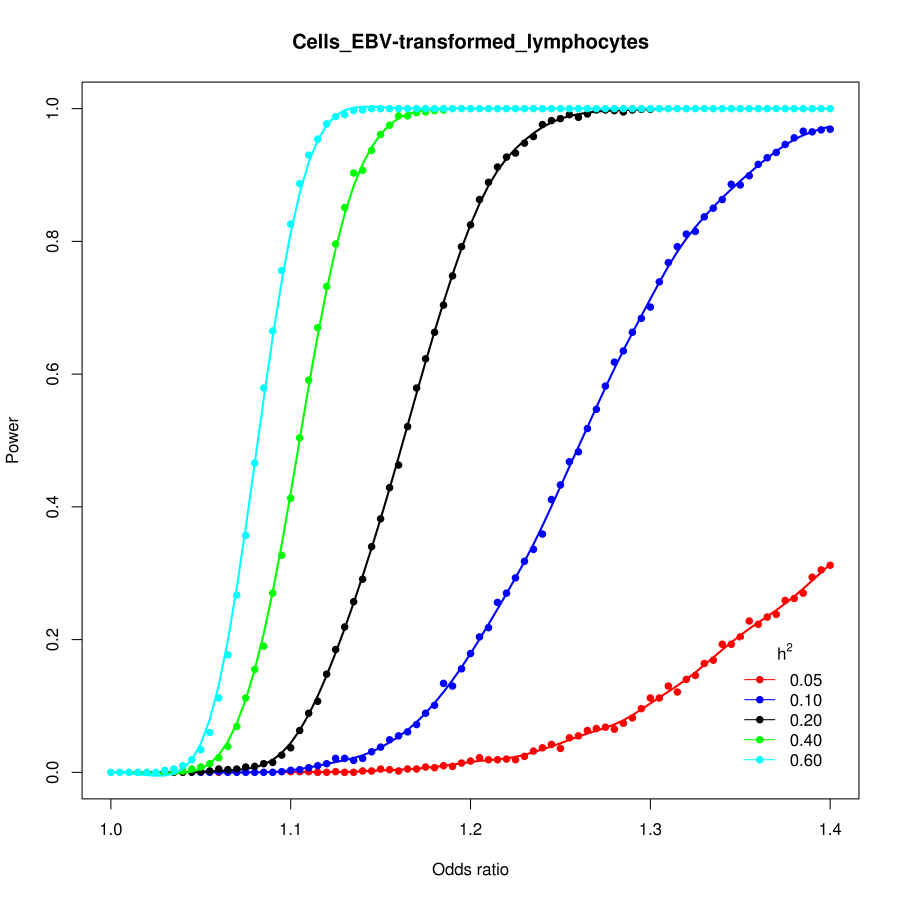
**

**Supplementary Figure 2: TWAS power plot in EBV-transformed lymphocytes.** Simulation analysis based on 7,319 cases and 234,385 controls. Gene expression was generated from the distribution of gene expression levels from EBV-transformed lymphocyte tissue. Statistical power was calculated at *P*<1.96×10^-6^ (the significance threshold used in the main TWAS analysis) according to various cis-heritability (*h^2^*) thresholds which are assumed to be equivalent to gene expression prediction models (*R^2^*). Power calculations were per 1 s.d. increase or decrease in gene expression based on 1,000 replicates.

**SUPPLEMENTARY REFERENCES**

1. Went, M. & Sud, A. Identification of multiple risk loci and regulatory mechanisms influencing susceptibility to multiple myeloma. **9**, 3707 (2018).

2. Broderick, P. *et al.* Common variation at 3p22.1 and 7p15.3 influences multiple myeloma risk. *Nat Genet* **44**, 58-61 (2011).

3. Chubb, D. *et al.* Common variation at 3q26.2, 6p21.33, 17p11.2 and 22q13.1 influences multiple myeloma risk. *Nat Genet* **45**, 1221-1225 (2013).

4. Mitchell, J.S. *et al.* Genome-wide association study identifies multiple susceptibility loci for multiple myeloma. *Nat Commun* **7**, 12050 (2016).

5. Swaminathan, B. *et al.* Variants in ELL2 influencing immunoglobulin levels associate with multiple myeloma. *Nat Commun* **6**, 7213 (2015).

6. Abecasis, G.R. *et al.* A map of human genome variation from population-scale sequencing. *Nature* **467**, 1061-73 (2010).
